# Machine Learning Integrating Protein Structure, Sequence, and Dynamics to Predict the Enzyme Activity of Bovine Enterokinase Variants

**DOI:** 10.1021/acs.jcim.3c00999

**Published:** 2024-02-22

**Authors:** Niccolo Alberto Elia Venanzi, Andrea Basciu, Attilio Vittorio Vargiu, Alexandros Kiparissides, Paul A. Dalby, Duygu Dikicioglu

**Affiliations:** † Department of Biochemical Engineering, University College London, Gower Street, WC1E 6BT London, U.K.; ‡ Department of Physics, University of Cagliari, Cittadella Universitaria, I-09042 Monserrato, Cagliari, Italy; § Department of Chemical Engineering, Aristotle University of Thessaloniki, 54 124 Thessaloniki, Greece

## Abstract

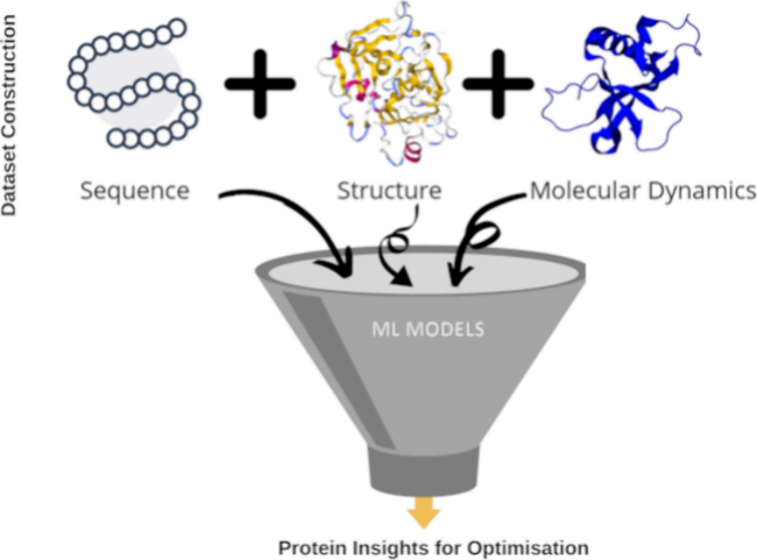

Despite recent advances in computational protein science, the dynamic behavior of proteins, which directly governs their biological activity, cannot be gleaned from sequence information alone. To overcome this challenge, we propose a framework that integrates the peptide sequence, protein structure, and protein dynamics descriptors into machine learning algorithms to enhance their predictive capabilities and achieve improved prediction of the protein variant function. The resulting machine learning pipeline integrates traditional sequence and structure information with molecular dynamics simulation data to predict the effects of multiple point mutations on the fold improvement of the activity of bovine enterokinase variants. This study highlights how the combination of structural and dynamic data can provide predictive insights into protein functionality and address protein engineering challenges in industrial contexts.

## Introduction

Proteins are essential, powerful machines in biology and consequently find a wide range of application areas in biotechnology, including the manufacturing of targeted therapies. However, their development as a functional product is expensive, time-consuming, and frequently yields unsuccessful results.^1^ The estimated average cost of bringing a new protein-based therapy to market is between $1 and $3 billion, and the success rate of clinical trials is below 10%. To overcome these obstacles, researchers are investigating new methods for expediting the engineering of proteins with enhanced functionality and manufacturability. Protein engineering entails making precise alterations to the original sequence of a protein to identify variants with desirable properties while minimizing interference with its function. Thus, protein engineering has revolutionized the production and use of protein-based products.^2^ However, due to the vast number of possible amino acid combinations, exhaustive experimental exploration of the landscape of protein fitness remains nearly impossible.^3−6^ Theoretically, the scope of mutations could be restricted to include only the ostensibly significant fragments of the protein such as the binding sites. However, this remains a heuristic solution applicable to a limited number of cases, as the majority of protein properties depend on the entire sequence and structural conformation, not just a few amino acids, due to the presence of epistatic effects.^7,8^

Biodescriptors are quantitative characteristics that shed light on a protein’s chemistry and structure. Algorithms can use the information contained in biodescriptors to predict the effects of amino acid substitutions on the properties of proteins. Recently, machine learning (ML) techniques have been applied to the classification of proteins and the prediction of the stability of protein–ligand complexes.^9−13^ Nevertheless, biodescriptors, which incorporate function-related properties associated with the macromolecules, were not employed in the investigation of the role of mutations introduced into the protein sequence on protein performance.^14−16^

Unsupervised ML methods mostly use natural language processing (NLP) to obtain sequence-level information for protein prediction. Recently, generative models that are able to design effective and diverse proteins suitable for various applications have received substantial attention, owing to their promise and potential. These models are all based on the assumption that sequence-based information, i.e., the order of the amino acids in a given length of peptide sequence, encompasses both structure and function information in its entirety. However, a true understanding of the physics and biochemical properties that go beyond sequence information has promise to be very useful for model interpretability and scientific discovery.

Molecular Dynamics (MD) simulations have emerged as an efficient method for describing a vast array of biomolecular processes, such as protein folding and molecular recognition, and for demonstrating how mutations affect the stability and function of proteins.^17−22^ MD simulation techniques allow the investigation of the conformational landscape of a protein and obtain direct information about its flexibility.^23−27^ Several approaches coupling ML algorithms with MD-derived data have been developed and successfully implemented over the past few years. MD-derived features were shown to be capable of predicting the stability of protein–protein and protein–ligand complexes^28^ and designing effective drug candidates for a known protein target.^29^ To our knowledge, however, there is no established or proposed method for predicting the effects of mutations on protein function by using machine learning algorithms that incorporate sequence-based information with MD simulations.

In this work, we present an ML workflow that leverages data generated via MD simulations with sequence- and structural-based features to predict the effects of multiple point mutations on the activity of an enzyme. The model protein employed in this study is bovine enterokinase, an enzyme used to remove affinity tags from high-value biopharmaceuticals.^30^ We applied a range of ML models to the 312 variants investigated in this study. The models utilized biodescriptors for sequence, structure, and dynamics-based features for each variant to predict its function and assessed the predictive performance of these models against empirical data available on the functional properties of each variant of the enzyme. We present here an effective machine learning-based strategy for incorporating different levels of information to successfully predict the functional properties of protein variants to enable faster and more powerful routes to protein engineering. We demonstrate the interpretability of these models by identifying the key biodescriptors contributing to the prediction of function and validate how the ML-based models can provide us key insight on the role of specific point mutations introduced to the protein sequence. We also discuss below the challenges and opportunities around incorporating simulation-based data as input for ML algorithms.

## Methods

### Experimental Data Set

This study used 312 variants of the engineered template bovine enterokinase (EKB), each containing one to nine mutations randomly introduced at the amino acid level in specific regions of the protein as described previously.^30^ The activity of the enzyme was determined upon expressing the protein at 30 °C with and without preincubation heating. The fold in activity was defined as the ratio of the activity of a variant to that of the template EKB. Here we intend to predict the difference between the two experimental settings measured, defined as the fold change in activities (FCA) (Table S1). The experimental data set was constructed by carrying out multiple rounds of error-prone PCR (epPCR), introducing modifications at the nucleotide level, resulting in a total of 312 variants containing zero to nine co-occurring mutations at the amino acid level.

### Homology Modeling and Variants Structures Constructions and Evaluations

SWISS-MODEL was used to build a template-based homology model of the engineered form of bovine enterokinase and the 312 variants (PDB ID: 1EKB).^31−33^ BLAST and HHBlits were used to search the SWISS-MODEL template library for the sequence of the engineered protein.^34,35^ Models were constructed with ProMod3 using the target template 1EKB.^36^ The final models were selected to maximize the Qualitative Model Energy Analysis Distance Constraint (QMEANDisco) and the Global Model Quality Estimation (GMQE) scores, evaluating both the stereochemical and energetic features together with structural similarity to 1EKB.^31,37^ AlphaFold-2 (AF2) was performed using one model structure generation with three recycles using amber and MMseqs2 (UniRef + Environmental) for Multiple Sequence Alignment (MSA) mode. The structures thus created were then compared to homology model structures using Biopython by superimposing and calculating the root-mean-square deviation per residue (RMSD).

### AlphaFold-2

AF2 was utilized by locally executing the publicly available ColabFold script. AF2 employs the predicted local distance difference test (pLDDT) to assess the accuracy of predicted C-alpha locations (on a scale of 0–100) with experimental structures as well as the predicted Template Modeling (pTM) to create projected aligned error (PAE) maps.^38−40^

### Molecular Dynamics Simulations

PROPKA’s^41^ online service was used to set the charge of the amino acids at the experimental pH of 8.0.^30^ The model was constructed on the GROMACS 2019.3^42^ molecular dynamics engine, and MD simulations were run using the OPLS-AA force field.^43^ TIP3P^44^ was used as the water model to allow faster computation. All of the proteins were placed in a cubic box, allowing for at least a 1.0 nm protein-edge distance in each dimension. The systems were neutralized using 50 mM Na^+^ and Cl^–^ ions randomly placed to resemble the experimental conditions under which the variants were expressed. The system was then energy minimized over a maximum of 50,000 steps, stopping at 1000 kJ mol^–1^ nm^–1^. Isothermal-isochoric equilibration at 300 K was performed over 100 ps using the leapfrog integrator with a coordinate update rate of 1.0 ps. The isothermal–isobaric equilibration was carried out using the same settings as the isothermal-isochoric step with the addition of a Parrinello-Rahman pressure coupling and a reference pressure of 1.0 bar. In all simulation steps, the standard Particle-Mesh-Ewald (PME) model^45^ was used to treat long-range electrostatic interactions in systems with periodic boundaries. A cutoff distance of 1.0 nm was set for short-range van der Waals and electrostatic interactions.

### Simulation Lengths and Evaluation of Variance Analysis among MD Repeats

A subset of 15 variants was randomly selected for the evaluation of the methodology before scaling the simulation protocol to the remaining variants. Following preliminary analysis, the 312 variant molecular structures were simulated five times at the selected length, leading to 1605 trajectories.

#### Statistical Analysis

A subset of 15 variants was selected for evaluating the methodology. These were simulated across 10 ns spaced simulation lengths ranging from 10 to 200 ns in triplicate. One-way Analysis of Variance (ANOVA)^46^ of the root-mean-square deviation (RMSD) values were used to assess the minimum simulation length and the variance between replicate trajectories. The simulation length comparisons focused on the last 10 ns of each simulation. The analysis was performed using SciPy 1.0 through the f_oneway function.^47^

#### Path Similarity Analysis (PSA)

PSA was performed using MDAnalysis.psa built-in function using Hausdorff distances.^48^ Hausdorff distance is an established metric used to compare the geometries of trajectories by comparing two paths *P* and *Q* as sequences of conformations. The distance is then calculated as
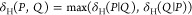
1where δ_H_(*P*|*Q*) is the directed Hausdorff distance from *P* to *Q*.
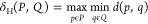
2

### Biodescriptors Data Set

A data set of 192 biodescriptors that provide information on protein sequence, structure, and dynamics was constructed for the 312 variants (Table S2).

#### Sequence and Structure Descriptors

The sequence embeddings were extracted using the R CRAN package “Peptide”, calculating global protein properties in the form of 66 features.^49^ These included: Cruciani properties,^50^ Kidera factors,^51^ zScales,^52^ FASGAI vectors,^53^ and the BLOSUM indices. Fourteen global properties of the variants were calculated using the module ProtParam of the Biophython package,^53^ including molecular weight and isoelectric point.

#### MD Descriptors

Root-Mean-Squared Deviation (RMSD) and Radius of Gyration (RoG) with respect to the initial structure were extracted for the following selections using the GROMACs package:^42^ whole proteins without non-hydrogenic atoms, the backbone, and Cα. These three features were also obtained for the binding site alone, defined as the residues within 3.5 Å of the ligand in the PDB crystal structure. The time series points were preprocessed, keeping the average and standard deviation in the final data set. The Dictionary of Protein Secondary Structure (DSSP)^54^ was also extracted as an indicator of structural characteristics of proteins throughout the trajectory, including parallel beta-sheets, antiparallel beta-sheets, alpha helices, 3–10 helices, turns, bent, and random coils using AMBER tools via Pytraj.^55^

#### MDpocket

MDpocket was used to extract additional features from the binding pocket calculated during the trajectory. The binding site was defined the same way as for the MD descriptors, where only residues within 3.5 Å of the peptide-like ligand in the template model were used for homology modeling. Features included the size and length of the binding site, its depth accessibility, hydrophobicity, the charge of each amino acid, the average number of times an amino acid was encountered in the binding site during the simulation, polarity/apolarity, and the total surface area as well as the normalized B-factor score of the binding site through the trajectory.^56^ For practical reasons, we refer to features in the rest of the text with acronyms, as defined in the Supporting Information in Table S2.

### Machine Learning Algorithms

The features were standardized using MinMaxScaler from Scikit-Learn.^57^ The data set was randomly split such that 80% was used for training and the remaining 20% was used as a test set. 42 supervised machine learning algorithms are present in Scikit-Learn (Table S3)^57^ and were fitted with their standard parameters to the data to find the best-performing template model to then carry forward for hyperparameter tuning according to Table S4. FCA was used as the response variable in the models. The models were implemented using the default parameters of Scikit-Learn and evaluated based on root-mean-square error (RMSE) (Eq. 3), *R*^2^ (Eq. 4), and execution time in seconds. Following the removal of those models with negative *R*^2^ from further consideration, the mean absolute error was added as a performance metric (MAE) (Eq. 5)
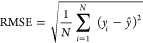
3
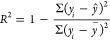
4
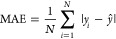
5where *ŷ* is the predicted value of *y*, *y* is the mean value of *y*. The normality tests were conducted by calculating the Fisher-Pearson correlation and graphically demonstrating the distribution of FCA with a Q-Q plot.

### Model Building

The performance of the selected algorithm, LightGBM, was evaluated when only specific categories of features were employed: sequence/structure descriptors (Seq), MD descriptors, or MDpocket descriptors, resulting in the following combinations: Seq+MD+MDpocket, Seq+MD, MD+MDpocket, Seq+MDpocket, Seq, MD, and MDpocket. All model parameters were tuned using RandomizedSearchCV from Scikit-Learn over 5 cross-validation folds through a defined search space with the negative mean absolute error as a scoring function (Tables S4 and S5). To evaluate the stability and variability of the model, we performed a total of 500 bootstrap iterations. During each iteration, a bootstrap sample was randomly selected with a replacement from the training set. The model was then trained using the optimal hyperparameters obtained by RandomizedSearchCV for each data set, and their performance metrics were calculated for the predictions made on the test set. ANOVA was then performed to determine if there are any statistically significant differences in performance across the different data sets. Subsequently, the Tukey Honestly Significant Difference (HSD) test was conducted to perform pairwise comparisons between the groups, identifying which specific data sets differ significantly from each other.

### Model Performance Evaluation and Analysis

The models built using the seven different feature combinations reported above were evaluated based on *R*^2^, MAE, and RMSE as defined in Scikit-Learn.^57^ The feature importance values were evaluated by using two methods: Tree SHapley Additive exPlanations (SHAP)^58^ and permutation feature importance.^59^ SHAP is a method based on game theory that assigns a value to each feature that represents its contribution to the prediction as well as providing insights on feature interactions. It computes Shapley values for each feature by averaging over all possible permutations of the features, resulting in an accurate measure of the feature importance. Permutation feature importance is a method that evaluates the impact of each feature by randomly permuting its values and measuring the resulting decrease or increase in the model’s performance, thus providing a direct measure of feature importance. Together, they can provide a comprehensive understanding of their features and their importance. Lastly, hypergeometric distribution testing was performed to identify patterns in the predictions.

## Results and Discussion

**Figure 1:**
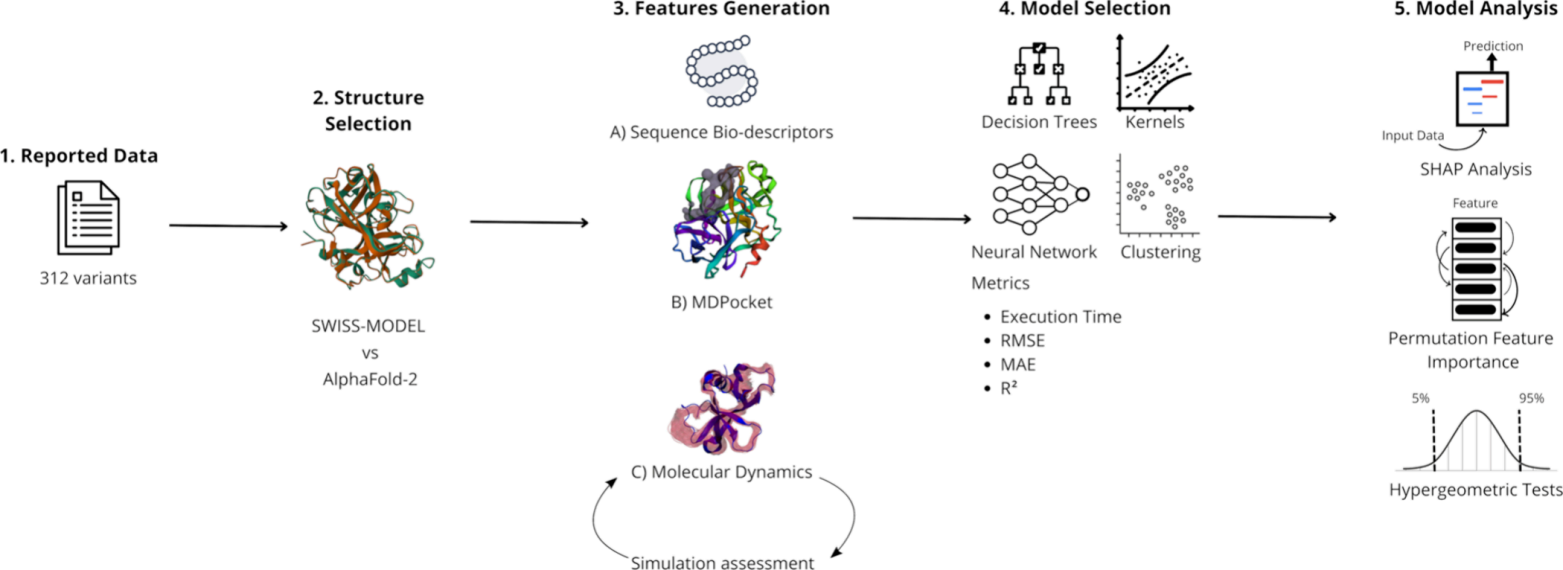
Proposed pipeline comprises MD simulation generated data with sequence and structure features for protein engineering on previously reported enterokinase bovine variants.

The aim of this work was to establish the role of dynamic features integrated with sequence and structure information in predicting protein function through ML. For this purpose, a case study containing 312 variants of the engineered Chinese Yellow bovine enterokinase light chain (EKL) was selected. The structures of all variants were predicted, and MD simulations were performed for all of the variants following the statistical evaluation of simulation lengths and the variability of the replicate simulations for each sample. The final data set was then constructed by extracting distinct categories of features, including sequence/structure and dynamics-based features, which were then used to predict FCA using various ML-based algorithms. The best-performing template model was then carried forward to assess the importance of features from different categories and those that contributed the most to the model’s performance (Figure 1). Each step of this newly proposed pipeline will be highlighted in the following sections.

**Figure 2:**
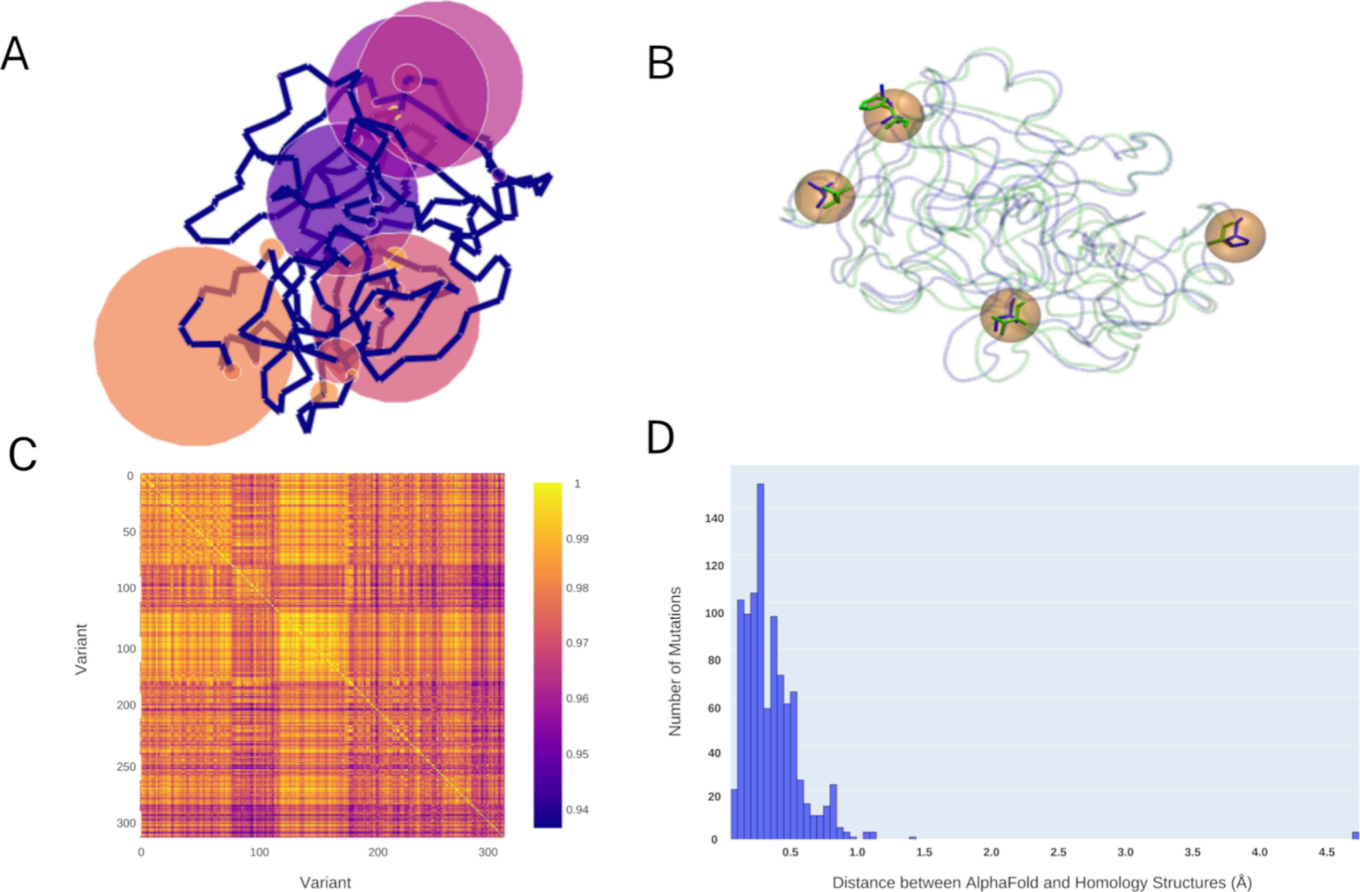
Construction of protein structures and data set descriptions. (A) A graph representing the enzyme’s mutation sites. The spheres are the normalized occurrences of mutations at specific sites throughout the data set. (B) Enterokinase bovine and engineered template enterokinase bovine superimposed with the mutation sites shown as licorice (PDB: 1EKB). (C) Heatmap of the identity matrix of the variants across the data set. (D) Histogram representing the RMSD between the mutations of the PDB constructed via AlphaFold-2 and the homology models.

Due to the nature of the epPCR protocol implemented, the data set comprised of mutations introduced only at specific sites across the protein structure, as depicted in Figure 2A. This methodology reflects the natural evolutionary pathway of the protein, resulting in a data set with similar amino acid content with an identity index above 90% and 29.7% of positions being at least mutated once through the data set (Figure 2B,C).^30^ Activities of the enzyme variants were measured with and without a heat-shock, and the performance of the variants was evaluated by a parameter that we call FCA here, which quantifies the difference in activities between the two experimental settings, with a high FCA indicating a measure of the extent to which the introduced mutations improved the protein’s activity.

### Building the Protein Structures

The enzyme for which variant enzyme activities were measured does not have a crystal structure available. This limitation was overcome by using the protein structure for the Yellow Bovine Enterokinase (PDB: 1EKB, resolution: 2.30 Å),^32,33^ the closest structure to the engineered enterokinase bovine with variants available. This structure and, consequently, the structures of the variants were required for the MD simulations and subsequent modeling steps. The difference between the two proteins is in the four mutation sites: V15Q/R82P/C112S/D176E (Figure 2B). To construct the mutations, we relied on homology modeling, as described earlier. The homology modeling offered quantitative and qualitative measurements for model assessment, resulting in an average GMQE and QMEANDisco of 0.88 and 0.86, respectively, and a standard deviation less than 0.01 in both cases.^37^ The process was repeated for the experimental variants, resulting in similar metrics across the 312 variants, which indicated the acquisition of rigorously proposed structures as indicated by these metrics.^37,60^ Since the initial protein structures are key elements of the proposed pipeline, it was essential to ensure that high-quality modeled PDB structures were made available before they were carried through the pipeline. For this purpose, an additional quality check was carried out to compare the structures generated via homology modeling to those proposed by AF2.

The pLDDT and pTM of the models obtained via AF2 were found to have an overall score above 90 and 0.90, respectively (Figure S1), in the variant sequence positions except for the N-termini, where the scores dropped below 80 and 0.80 in all of the models. Upon obtaining the AF2 models, the point mutations of the homology structures were compared with those of the AF2 generated ones, with 79% of the mutations displaying close similarity with an RMSD of 0.50 Å and 99% presenting an RMSD below 1.00 Å. The differences in residues were discarded if the distance was determined to be 1.00 Å, as these relate to conformational differences between the amino acids, also indicating possible manifestations of variations during the energy minimization step of the MD simulations. The presence of residues with more than a 1.00 Å difference was recognized as an indication of the fact that both the backbone and the rotamers of the amino acids have been modeled differently. These include mutations in the data set at the sequence positions 47, 49, 83, and 95 with RMSDs of 4.75–4.79, 1.45, 1.00–1.06, and 1.12–1.15 Å, respectively. A closer look at the secondary structure of these sites showed that positions 47 and 49 are part of a loop, whereas positions 83 and 85 belong to a random coil conformation. This finding is in accordance with previously reported findings highlighting AF2’s inability to model intrinsically disordered regions, which led to the decision to select the homology model structures for the simulations.^61−63^

### Understanding the Role of Simulation Length and Robustness in MD Analysis

The pipeline development sought (i) to determine the minimum informative MD simulation length ranging from 10 to 200 ns and (ii) to evaluate the extent of variability between repeat simulations of the same case. Prior to running 312 computationally costly 200 ns simulations for all variants with replicates, we randomly selected a subset of fifteen variants with an FCA that was statistically representative of the whole data set (two-tailed Welch-corrected test, *p*-value = 0.78).

#### Minimum Informative MD Simulation Length

Conducting the MD simulations at the scale proposed here, which comprises more than 1600 trajectories, when considered in conjunction with the possibility of employing the pipeline in studies of even larger scale, necessitates a careful evaluation of the simulation times for the MD simulations. Therefore, the tradeoff between the information gained with extended timeframes and the computational cost of the extended simulation times needed to be carefully evaluated. For this purpose, the effect of simulation lengths on model success parameters was investigated by running three replicates of the simulations for a randomly selected subset of fifteen variants, which were simulated for extended times of up to 200 ns, and the trajectories were investigated in 10 ns increments starting from the shortest trajectory of 10 ns. As RMSD had the highest variance among the MD-based features extracted in this study, it was selected as the main feature for the statistical evaluation of the trajectories.^64^

**Figure 3:**
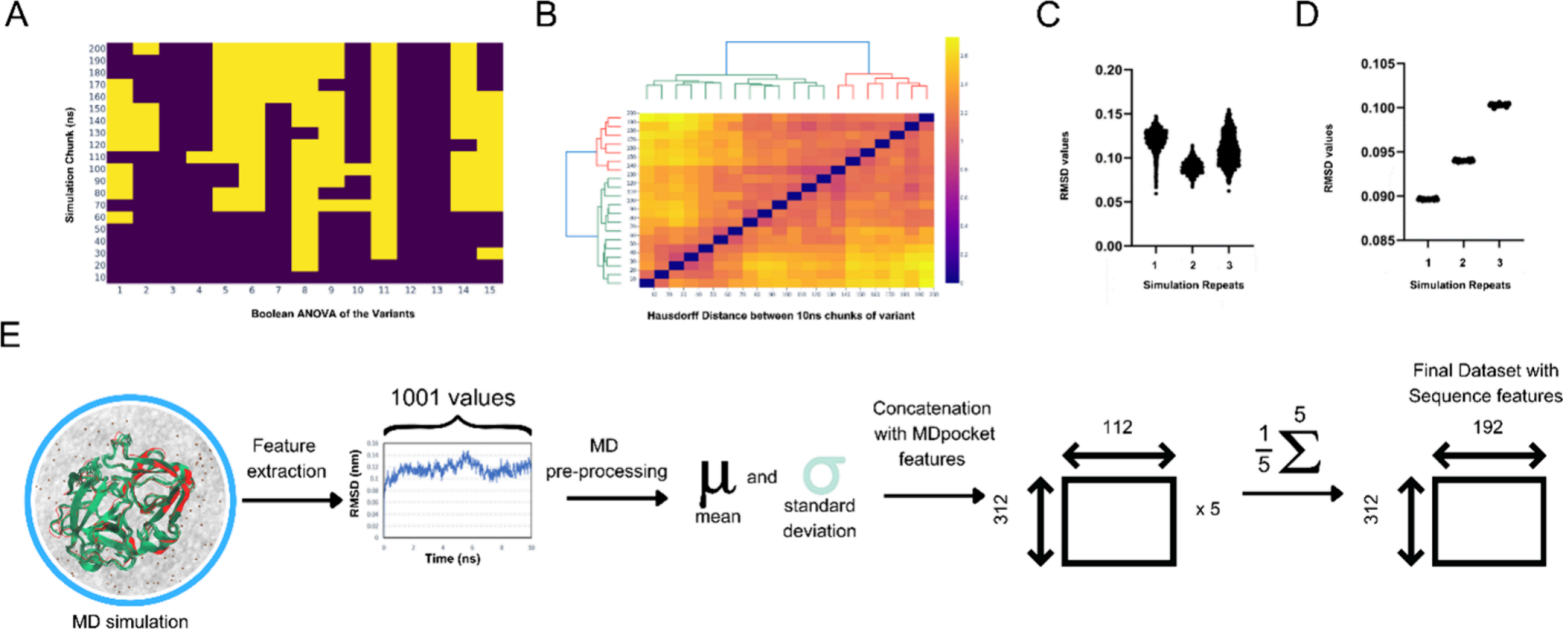
Statistical evaluation of the MD simulations. (A) ANOVA heatmap showing statistical differences across the variants up to 200 ns for 10 ns chunks against the first 10 ns (keys: purple = non statistical difference, yellow = statistically different). (B) Heatmap of Hausdorff distances with ward dendrogram showing clusters and distances between 10 ns simulation chunks across a trajectory. (C) Violin plot of the RMSD values of the simulations for a randomly selected variant from the 15-variant subset. (D) Individual dots plot for each element of the 20-cross fold averages of the same randomly selected simulation shown in (C), confirming the preservation of the differences between repeats of the simulation of the same protein structure after averaging. (E) Through statistical evaluations, the dimension of the final data set was reduced to 112 × 312 (MDpocket features × number of variants) from the initial set of 80 × 5 × 32(1001) × 312 (number MDpocket × replicate trajectories × MD-based biodescriptor entries per trajectory × number of variants). These were then concatenated with 80 sequence and structure biodescriptors, resulting in a data set of 192 features by 312 variants.

A number of different approaches were selected to carry out this evaluation, as the findings emerging from the analysis would lead to decisions that would impact the success of the predictions made by the machine-learning models. A method that uses Principal Component Analysis (PCA) was previously proposed to assist decision-making around the similarity between trajectories; however, PCA of this data set did not yield any comparative insight as to the differences observed between trajectories run at different lengths, and therefore, more suitable alternative methods were employed to make conclusive decisions.^65^ ANOVA was previously shown to work satisfactorily in comparing MD trajectories.^66^ In our analysis, there was no statistically significant difference in the RMSD values obtained from a 10 or a 50 ns trajectory for 80% of the randomly selected variants investigated here. However, statistical differences were observed when the RMSD of the first 10 ns sets were compared with that for trajectories longer than 50 ns. On the other hand, above 50 ns, the simulations were observed not to be significantly different from one another (Figures 3A and S2). While longer simulation times would be essential to capture the dynamic behaviors during large conformational changes, in the case of a compact globular protein, such as that of enterokinase, it was a reasonable assumption that the nature of the dynamics of the protein could be captured by small conformational changes captured at shorter simulation timeframes.^67^

As an alternative to the statistical evaluation of the RMSD values, PSA was employed to compare the 10 ns simulation chunks to 200 ns. PSA enables a quantifiable similarity metric, Hausdorff distance (δ_H_) of the different paths identified during an MD simulation. The distance can be used to measure the similarity between the paths and thus provide a metric of comparison between different simulation lengths. We partitioned the trajectories into separate trajectory files of 10 ns each and determined the PSA Hausdorff distances on the data. The simulation subtrajectories had relatively small Hausdorff values, with a maximum δ_H_ of 2.16 across the triplicates of the 15 simulation sets (totaling 45 sets) (Figure 3B). This small difference between the simulation segments implies a high similarity between the simulation trajectories based on previous reports where trajectories with path lengths of δ_H_ < 0.5 Å were denoted as identical and δ_H_ > 3.0 Å as highly different.^68^ The similarity of the time segments for each trajectory indicates the similarity in the features to be extracted from these trajectories. This analysis, in conjunction with the statistical evaluation and the globular and compact nature of the bovine enterokinase enzyme used in this study, provided convincing evidence for us to select the first 10 ns of the simulation trajectory to perform further analysis. Consequently, this would allow a reasonable computational time frame within which the MD data set would be generated.

#### Degree of Variability between Simulation Repetitions

For the ANOVA study, the RMSDs of the MD trajectories were observed to vary significantly between replicates, which is in accordance with previous reports where low *p*-values were reported.^69,70^ For MD simulations run over the course of a fixed time frame, correlations in biodescriptors presented within a given time segment were previously shown not to cause statistically significant differences, leading to vanishing *p*-values.^71^ In line with this observation, the averaging of the MD biodescriptors over a given time segment was proposed.^29,72^ Such an approach was implemented in other studies when working with MD simulation data.^29^ However, it should be considered that averaging may possibly offset the inherent variation in the data. In order to assess this further, we performed a 20-fold validation to evaluate if the variation embedded in the simulations was retained upon averaging. 80% of the trajectory data points were randomly shuffled, and the RMSD values were thus calculated. These average values remained statistically significant between trajectories (Figure 3C,D) and thus retained the variability of the trajectories within a protein variant. After the significant variation across different trajectories was maintained upon averaging to retain crucial information that can be utilized by ML algorithms, a decision was made to move forward with the averaged values for the MD features.

Since a significant variation between repeated simulations of the same protein model was shown to persist even after averaging the trajectories, a decision was made to increase the number of repeat simulations from three to five with the aim of capturing the inherent variability of the simulation space adequately and effectively, in line with former practice reported.^70^ All models were constructed using the average and standard deviation of the averages of the biodescriptors for five trajectories from this point forward (Figure 3E). This ingestion of MD features was shown to be capable of capturing key features linked to protein function and was shown to be a suitable decision for the purposes of the model-based analysis discussed below.^29^

### Selection of a Suitable Machine Learning Model

The final data set of 192 observed variables denoted as biodescriptors was used to evaluate the predictive power of 41 machine-learning models for the 312 variants under investigation (Table S5). We applied an extensive range of learning algorithms, including Ensemble models (Decision Trees), Single-Tree-based models, Gaussian Processes, Linear Regression, Clustering Regression, Neural Networks, and Kernel-based approaches. A comprehensive list and description of the models are available in Table S3. We opted to conduct a preliminary screening for model selection using the default model parameters available in scikit-learn or their packages (e.g., XGBoost). This decision to use default parameters to identify the algorithms to be carried forward is in accordance with the consensus that there is no preferable model *a priori*.^73^ The models were first ranked and screened according to three metrics: *R*^2^, Root Mean Square Error (RMSE), and time taken in seconds for the execution of the algorithm, as calculated using scikit-learn functions.

Seven of the models tested; RANASCRegressor, Linear Regression, TransformedTargetRegressor, ExtraTreeRegressor, GaussianProcessRegressor, KernelRidge, and PassiveAggressiveRegressor, produced negative values for the coefficient of regression, indicating that these models fit the data worse than a horizontal line would do; in other words, those models were only predicting the average value of the input variables. Consequently, these models were excluded from further consideration. The FCA probability density function manifested a normal-like skewness (0.16) and kurtosis (0.45) in its distribution (Figure S3). The Poisson and Gamma Regression algorithms did not fit the distribution of the response variable, a prerequisite for the models’ training, and they were thus excluded from further consideration.

**Figure 4:**
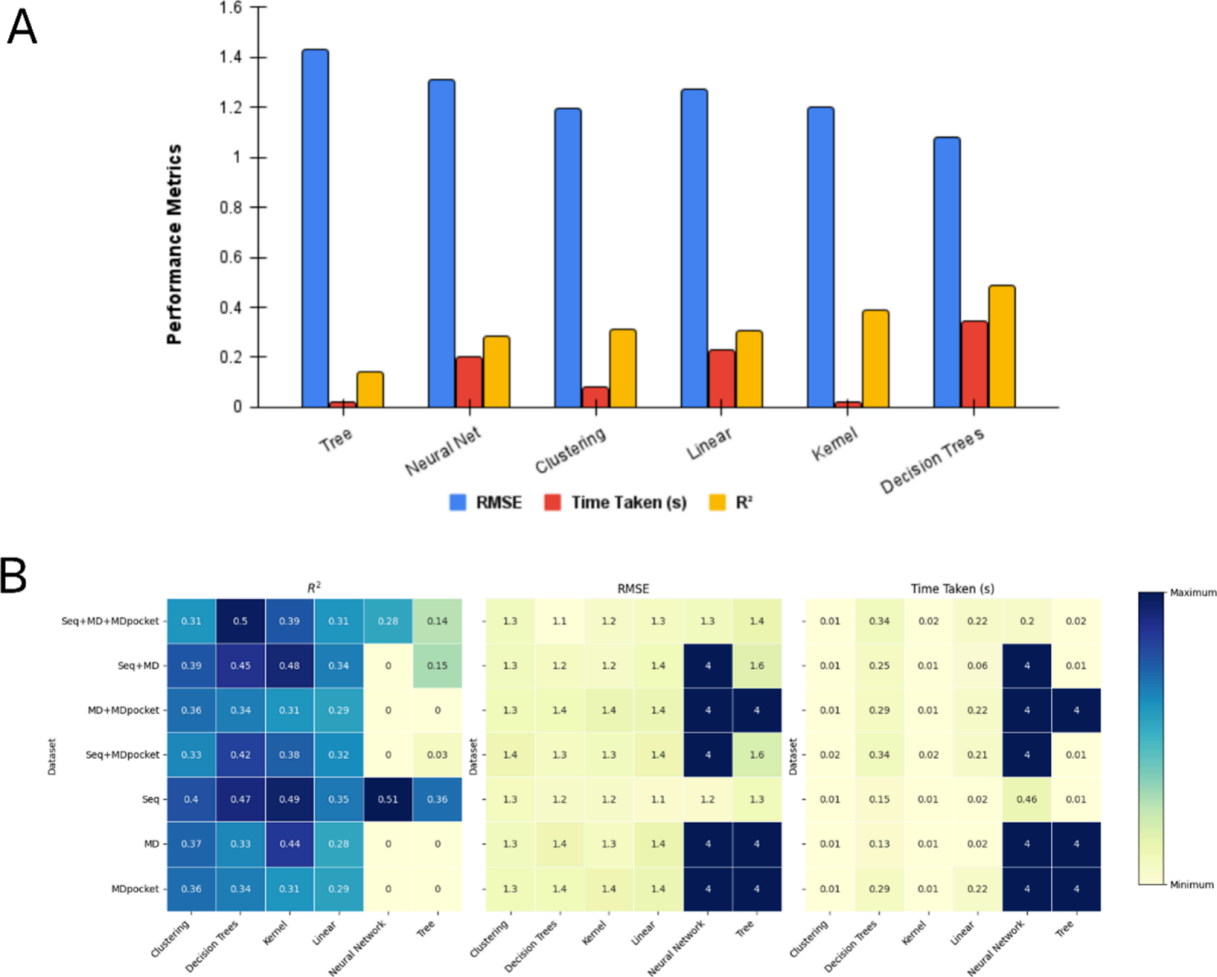
Performance evaluation of the ML models for predicting FCA employing all features from the three biodescriptor categories Seq, MD, and MDpocket. (A) Average performance of the algorithm classes. (B) Heatmap of averaged performance (scaled from 0 to 1) of the algorithm classes when features from specific biodescriptor categories were employed: Seq Seq+MD+MDpocket, Seq+MD, MD+MDpocket, Seq+MDpocket, Seq, MD, and MDpocket.

The predictive power of the remaining 32 models was assessed by comparing the predicted and empirical values of the response variable, FCA, for variants of the engineered template form of the bovine enterokinase light chain in the test set. Despite their generally longer runtime compared to other algorithm types, decision trees provide substantially improved model fit with an average unadjusted *R*^2^ score of 0.48. This was 0.10 higher than the *R*^2^ score of the second-best performing class of algorithms, as indicated in Figure 4A. We further performed an analysis for the decomposed data sets that contained features drawn exclusively from the following biodescriptor categories in order to evaluate whether any of the model classes would perform superior for data originating from specific biodescriptor categories: Seq+MD+MDpocket, Seq+MD, MD+MDpocket, Seq+MDpocket, and Seq, MD, and MDpocket. The decision tree-based models yielded the lowest RMSE across all seven data sets as well as ranking high for performance as indicated by *R*^2^ (Figure 4B). This evaluation conclusively showed that the decision tree-based algorithms did not exhibit any bias for modeling a particular group of features while maintaining high algorithm performance.

Among the decision trees tested, the Gradient Boosting Regressor (GBR) generated the resulting models with the lowest RMSE on the Seq+MD+MDpocket data set (Figure S4). Although GBR was praised as a regressor that allowed for a high degree of generalizability and interpretability,^74^ GBR’s trees are constructed based on the surrogate loss function for minimizing the error of the overall model. As such, this function can only be considered a proxy for the true loss.^75^ However, the Light Gradient Boosting Machine (LightGBM) algorithm calculates the second-order derivative of the loss function. This alternative approach was reported to allow for a quick and accurate minimum search of the loss function.^76^ LightGBM offers several advantages over other decision trees: (i) its regularization is more complex, thereby preventing overfitting; (ii) it admits sparse features and offers interpretable tools for model analysis; and (iii) it retains performance in high-dimensional data sets.^75,77−79^ Lastly, previous reports have shown that LightGBM improved the model’s generalizability.^80^ In our analysis, the difference between GBR and LightGBM in RMSE was only 3%, and in the current investigation, LightGBM ran noticeably faster than GBR (Figure S4), taking 0.05 compared to 0.38 s. All such advantages led to the decision to select LightGBM to conduct the downstream modeling for predicting FCA in this work. The hyper-parameter tuning of the models was carried out as detailed in the Methods section.

### Evaluation of Model Performance

The performance of the models was evaluated based on three metrics: *R*^2^, RMSE, and MAE. RMSE and MAE provided measures of the difference between the observed values and those predicted by the estimator. The evaluation was performed on hyperparameter-tuned LightGBM decompositions of the data sets iteratively to assess the dependence of the model on specific features. The hyperparameters for each data set are presented in Table S6.

Applying the algorithm to MD and MDPocket features only, we noticed strikingly low *R*^2^ values (Table 1) implying that there was not a strong correlation between these features alone and the predictions made on the performance of the enzyme variant. Since the MD features extracted here are not meaningful unless associated with structural and sequential information, the poor predictive performance would be unsurprising. Furthermore, the limited number of MD features (32 as opposed to 80 sequence-based and structure-based biodescriptors) rendered building models with high predictive capability difficult given the unbalanced number of feature classes in the data set. This hypothesis was further supported and confirmed by the observation that the inclusion of the other two subsets of features together with MD-based biodescriptors remarkably improved prediction accuracy.

**Table 1:** Key Performance Indicators Evaluating the Predictive Capability of the Models That Used Features from Specific Sets of Bio-Descriptor Categories^a^

Feature Combination	*R*^2^	RMSE	MAE	Approximate time required for data generation per variant
**Seq+MD**	**0.50**	**1.18**	**0.83**	45 min
**Seq**	0.47	1.21	0.86	5 s
**Seq+MD+MDpocket**	0.45	1.24	0.86	2 h
**Seq+MDpocket**	0.42	1.27	0.86	2 h
**MD+MDpocket**	0.21	1.48	1.08	2 h
**MD**	0.22	1.47	1.08	45 min
**MDpocket**	0.20	1.49	1.16	2 h

a Rank of the models that utilized different feature combinations based on the different performance metrics is provided in brackets for the 63 cases of the 312 variants in the test set. In bold: the best-performing feature combination and underlined: the second best.

The models that were trained solely on sequence-based features had the second-best RMSE and MAE values. The predictive accuracy of the models improved when sequence features were used in conjunction with MD features across all metrics assessed here assessed: *R*^2^ (ca. 6.4%), RMSE (ca. 2.5%), and MAE (ca. 3.5%) representing higher predictive capabilities (Table 1). However, such performance differences are small and might be influenced by factors such as the choice of random seed or the limited data set size (312 variants in this study). To address these concerns and assess the model robustness, we performed 500 bootstrap iterations with replacement. There were no significant statistical differences between the Seq and Seq+MD data set across all metrics (Tukey HSD results between Seq and Seq+MD with a threshold of 0.05: *R*^2^*p*-value = 0.941, RMSE *p*-value = 0.9081, MAE *p*-value = 0.9844) (Figure S5). However, both data sets showed statistical superiority over the remaining four data sets. Given this finding, the Seq+MD model, which integrates sequence- and structure-based features with MD-based features, was selected for subsequent analyses. This decision was driven by the potential for richer insights from the added features that capture the system’s dynamics and thus improve the explainability or the interpretability of the models.

### Feature Analysis and Feature Importance

Machine learning algorithms were shown to be highly effective in depicting the data landscape they are exposed to.^6,81^ This ability to translate a data landscape description into an understanding of the relationships between observable and response variables is of utmost importance in protein engineering, where the connection between protein function and product creation is crucial.^6^ Therefore, it is imperative to develop an interpretable system that can use the information gathered by these models. The literature offers various ways to measure the value of a feature, and for this work, we employed two methodologies, permutation feature importance and SHAP analysis.^82^

**Figure 5:**
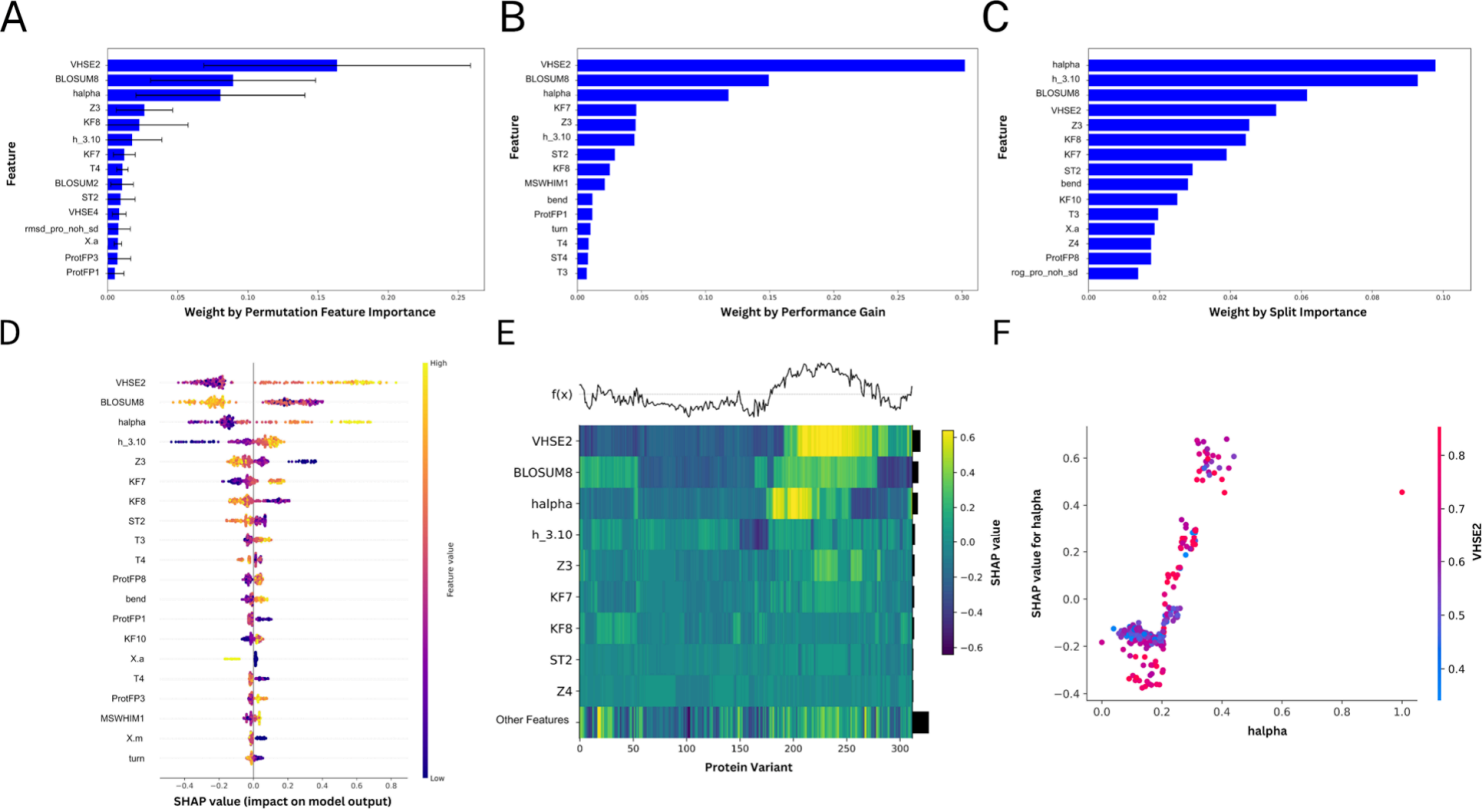
Ranking of features according to the Seq+MD data set using Permutation feature importance and SHAP. (A) Permutation feature importance ranking. (B) Features ranked by the performance gain they provided. (C) Features ranked by the number of times each feature was used to make splits in the data. (D) Feature importance bee swarm plot ranking features according to their impact on model output measured via SHAP. Each dot represents the value of a datapoint in the data set and is colored according to the feature value. The SHAP value related to each datapoint is a measure of how much knowing that datapoint affected the prediction. Negative and positive SHAP values represent, respectively, a decrease and an increase in the predicted value. (E) Top: the function of the model performance (*f*(*x*) horizontal line being the true FAC and the oscillating line the predicted value per instance). Bottom: Heatmap of the features according to their SHAP values showing how differently they altered the predictions across the data set. (F) h-alpha dependence plot showing the distribution of the SHAP values against the actual h-alpha values. The coloring is based on a second feature, in this case, VHSE2, with the strongest interaction effect.

Permutation feature importance is a highly effective method that plays a pivotal role in identifying the tangible contributions of individual features to the overall performance of a model.^83^ This algorithm is designed to break down the complex relationship between various characteristics and their impact on outcomes. By doing so, it provides an accurate and realistic landscape of how each feature contributes to the overall performance of the model. The final model was identified to be heavily dependent upon a specific set of features, including DSSP-derived features such as h-alpha as well as a range of other MD dynamic and sequence-based features from BLOSSUM, VHSE, and zScales (Figure 5A–F). These features were found to be instrumental in predicting FCA. The analysis identified unique connections between features such as the helix composition, the protein’s hydrophobicity, and FCA. This discovery can be used as a guiding point for protein engineering research in the future, providing invaluable insights into the development of more effective models. The machine learning modeling of the enzyme variant data showed that the selected model was able to learn from both sequence-based and molecular dynamics-based features. When the model relied solely on MD-based features, it detected a subset of MD features that were different from those used in models that integrated MD descriptors with sequence-based descriptors (Table S7). In the latter scenario, additional features were identified as important, such as the fraction of protein with random coil patterns throughout all of the simulated trajectories. This phenomenon, known as feature domain alteration, illustrated how a single MD-based feature can be contextualized when supplemented with sequence-based features as model descriptors. This is because decision trees, which are used by the model employed in this work, can facilitate or disrupt innovative feature interactions as required, allowing different characteristics to be identified as important for accurately representing a data set. It should be noted that the sequence-based descriptors used in this study were not position-based descriptors based on the alignment of the mutant sequences such as one-hot descriptors. One-hot encoding, as a descriptor, could implicitly capture some 3D structure information and thus lead to predictive capability comparable to that of the models that included the MD-based features; they do not universally offer the interpretability we sought after in this study to get meaningful insights for subsequent rounds of protein engineering. By using interpretable features contributed by the MD-data set, we aimed to facilitate a deeper understanding of the underlying mechanisms and guide future engineering efforts through feature importance.

To delve deeper into the impact of different descriptors on the algorithm’s decision-making, we modified the ranking criterion from permutation to performance gain and the importance of a feature in the algorithm’s decision to split the data set into branches (Figure 5A–C). This in-depth investigation showed that the features that contributed to the formation of decision trees predominantly stemmed from MD features, specifically those related to the binding site, the proteins positioning relative to the backbone and radius of gyration, random coil conformations, and the para- and beta-sheet conformations. Although it is unlikely that the MD characteristics were the primary contributors to the model’s numerical performance, as we deduced from the poor predictive performance of models that relied solely on MD-based data (Table 1), they played an important role in the algorithm’s decision-making process that ultimately led to a successful prediction of the output response. Consequently, the predictive capability of the models that incorporated MD-based features was greatly enhanced.

After completing the analysis of the model’s features using permutation feature importance, we proceeded to employ the SHAP methodology to compare and further evaluate the obtained results. This method was specifically designed to provide an in-depth explanation of individual predictions, offer a comprehensive overview of the model’s overall performance, and feature interactions. Notably, over 70% of the features identified as the 20 most influential 20 features contributing to the model’s decision-making by permutation feature importance were also identified as the most influential features by SHAP. This discovery confirms the findings through an independent algorithm-based search, as demonstrated by permutation feature importance.

Furthermore, the use of SHAP facilitated the establishment of a sense of concurrence between the response variable and the features, enabling the identification of how these biodescriptors were employed for each prediction. Upon analysis, it was discovered that higher percentages of amino acids under helices conformation, specifically h-alpha and 3.10 helices (h_3.10) (Figure 5D), were found to be related to higher FCA values. Conversely, the prevalence of turn conformations in the protein was demonstrated to have a negative influence on the FCA of the specific variation. This finding suggests that features representing both h-alpha helices and turns conformations were identified as key features, implying that the number of turns may have worked as a control system to reduce the number of reversals in the protein structure. It is worth noting that SHAP enabled the identification of essential aspects in MD that are frequently overlooked as well as the explanation of how these features were related to enzymatic properties, in this case FCA. Overall, this approach provided an in-depth and comprehensive understanding of the relationship between specific features and their impact on the response variable, shedding light on the underlying mechanisms that govern enzymatic properties.

Further, SHAP analysis identified several variables that have an impact on increasing or decreasing the predicted FCA values (Figure 5E). In Figure 5E, it is possible to evaluate each individual prediction and how the features were aggregated to construct the predicted values, as shown by the *f*(*x*) showing as a horizontal line the true value and the splines representing the predicted values. Notably, the variants with the largest amount of absolute error are found to be between 200 and 275. Particularly for these sets of variants, VHSE2, BLOSUM8, and h-alpha present large SHAP values, meaning that they contribute extensively to increasing the predicted value of FCA. The magnitude of this impact prompted us to investigate further to see whether there were any potential synergies for the output predictions. Specifically, we examined whether there were any interactions between cognate features with high SHAP values that could affect the accuracy of our predictions. After scrutiny, we found that h-alpha was a potential candidate for such interactions, having strong feature interactions with VHSE2, the second most informative feature (Figure 5F). This finding emphasizes the close correlation between sequence-based and MD-based biodescriptors, highlighting the importance of considering both types of descriptors in predictive models.

### Hypergeometric Testing

Hypergeometric testing is a statistical method used to determine the statistical significance of observing a certain number of successes (*k*) out of a sample size (*s*) within a larger population size (*N*), given that the population contains a total number of occurrences (*K*) with the specific characteristic being studied.^84^ In other words, it determines if a sample is random or whether it over- or under-represents a specific population. It is commonly employed in bioinformatics, but it has been applied in other areas of research and analysis.^85^

In the context of model performance analysis, hypergeometric testing can be used to assess the significance of the overlap between the predicted and actual outcomes. It is particularly useful for testing categorization models to assign instances to predefined classes or categories. To facilitate a comprehensive analysis of model performance, we propose a threshold-based approach to assigning labels to predictions. By calculating the threshold at 95% and 5% of the distribution of the difference between the predicted values and the true values, we were able to identify poor predictions beyond these thresholds. This label assignment process enables a fine-grained evaluation of the model performance of a regression task with classification labels.

We utilized this analysis to investigate whether there was any underlying bias created by the number of mutations introduced into the variants that would impact the predictive capability of this modeling pipeline. By constructing a contingency table capturing the counts of observed and predicted outcomes, we calculate the *p*-value representing the likelihood of observing at least as much overlap as that observed under the null hypothesis of random predictions. A significance threshold of 0.05 was set to determine the statistical significance. In the poor prediction’s thresholds, we identified *s* = 6 with *k* = 4 occurrences having the number of co-occurring mutations ≥5. According to the hypergeometric distribution testing, the occurrences in the poor thresholds were over-enriched by 3-fold compared to expectations, with a hypergeometric *p*-value of 0.01. This *p*-value indicates the statistical significance of the observed overlap between the predicted and actual outcomes. In this context, having the obtained hypergeometric *p*-value below 0.05 is considered statistically significant. This finding implies that our model has worse performance when predicting the effects of five or more co-occurring mutations, as these are over-enriched observations in the poor predictions. While it was an informative observation, this finding could have been biased since only 24.7% of the data contains more than five mutations, rendering this group under-represented within the data set. On the other hand, the relative scarcity of variants with a high number of mutations could have been caused by the enhancement of undesirable modifications leading to problems in protein folding or function. We further investigated these variants with a high number of mutations and identified that three of the six poor-performing predictions were the sole cases where the predictions were above the 95% threshold and that these variants shared the same four out of five mutations: S38T/L74F/M100K/S127T. As these mutations occurred in only 9% of the variants across the data set, we believe that integrating more data with variants containing large mutation counts could aid in better model performances and provide further assistance to guide improved advice for direct mutagenesis strategies in protein engineering.

## Conclusions

In this work, we present a machine learning-based modeling pipeline that integrates sequence-based and structure-based protein features with dynamics-based features extracted from large-scale data generated by MD simulations to predict protein functionality. The framework addresses some of the current challenges and limitations in protein engineering, particularly those around the identification and prediction of subtle differences between variants of the same protein. We showed that the approach proposed here performed successfully with sufficient predictive power to guide the engineering of novel protein designs and to provide mechanistic insight into the functionality of the protein. The pipeline can allow novel hypotheses to be derived from within a large search space in a feasible time frame to recommend the design of proteins equipped with desirable properties, which can then be realized through site-directed mutagenesis with minimal experimental effort.

Utilizing an information-driven approach to interpret ML-based models, we demonstrated that the information provided by MD-derived, highlighted the essential role protein dynamics plays in predicting function prediction. We highlight the following key attributes of this pipeline development process to guide future efforts in this domain. This study is the first of its kind to integrate replicate MD trajectories into an ML processing pipeline for predicting mutation effects on protein functions, and as such, we were able to highlight some challenges that are inherent in MD data sets. Through statistical analysis, MD simulations were shown to be very noisy, which necessitated dedicated evaluation and preprocessing prior to their integration within the ML pipelines. Model screening highlighted the superior ability of decision tree algorithms to exploit high-dimensional data sets for protein function prediction tasks. Decision trees yielded models that allowed for an interpretable understanding of the predictions.

The relative dominance of alpha helices in protein conformation was identified as a key characteristic impacting the models’ predictive performance for the EKB protein. This characteristic, represented by the feature h-alpha, is often overlooked and thus not evaluated during MD analysis. As we demonstrated here, such findings can allow a preassessment of protein engineering features, and using this pipeline, we can identify features from MD that can consequently be used to propose rational designs for protein engineering, possibly substituting residues that favor α helix formation.

One of the most important challenges to the successful implementation of ML-based approaches lies in accessing high-quality data. In the proposed pipeline, MD simulations were coupled with sequences to generate the input data set of protein features. Despite the limited availability of experimental data still being a bottleneck, for MD simulations the outlook is far from bleak, with the increasing availability of protein structures reported through crystallography and more recently in silico methods such as AlphaFold and the development of AI-based MD force fields.^86^ Furthermore, advancements in computing power and resources will allow for the extension of the applicability of the proposed protocol.^87^ The improvement in the data sets reinforced through MD will inevitably increase the predictive power of approaches such as the pipeline presented here, which will consequently boost efforts toward protein engineering in healthcare applications and in sustainable manufacturing.

## Data Availability

The raw data is available as Supporting Information Tables S1 and S5 at https://zenodo.org/records/10511492. Larger data, such as raw MD simulaitons (>10TB) and ML models, are available upon request. The relevant scripts are publicly shared via GitHub: https://github.com/NAEV95/Engineering_EKB.

## Supporting Information Available

Examples of AlphaFold-2 protein structure models, description of ML algorithms and settings, the preprocessed data, and further figures showing ANOVA analysis details and data insights (PDF)
