# Supplementary material for: Machine Learning Integrating Protein Structure, Sequence, and Dynamics to Predict the Enzyme Activity of Bovine Enterokinase Variants
Source: J Chem Inf Model. 2024 Feb 22;64(7):2681–94. doi: 10.1021/acs.jcim.3c00999 (PMC11005043; doi:10.1021/acs.jcim.3c00999)
Supplement: Supplementary file 1 — ci3c00999_si_001.pdf [file ci3c00999_si_001.pdf]

Supporting Information: Machine learning integrating protein structure, sequence, and dynamics to predict the enzyme activity of bovine enterokinase variants

*Niccolo Alberto Elia Venanzi[1], Andrea Basciu[2], Attilio Vittorio Vargiu[2], Alexandros Kiparissides[1,3], Paul A. Dalby[1], Duygu Dikicioglu[1]**

1. Department of Biochemical Engineering, University College London, Gower Street, London WC1E 6BT, UK

2. Department of Physics, University of Cagliari, Cittadella Universitaria, I-09042 Monserrato (CA), Italy

3. Department of Chemical Engineering, Aristotle University of Thessaloniki, 54 124, Greece

*Corresponding author.

Corresponding address: d.dikicioglu@ucl.ac.uk

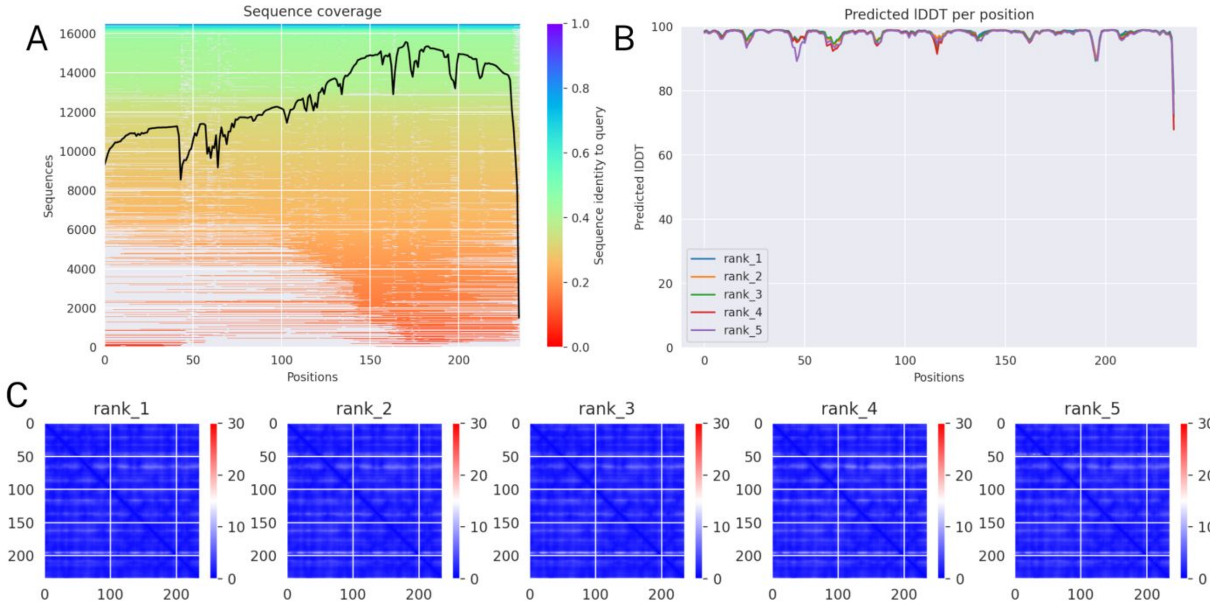

Figure S1. Alpha-Fold 2 example plots on the generated structures for evaluation. Here we show just one example. A) Multiple Sequence Alignment Coverage (MSA) seems excellent except for the N-terminus. Overall, it has a high number of sequences (mostly > 10000). B) Confidence in the prediction, measured via pLDDT show high accuracy in all the 5 models generated per protein. (C) Predicted Alignment Error (PAE) gives an estimate of the relative position of domains. In all the ranked models the error is below 10, implying high quality structures.

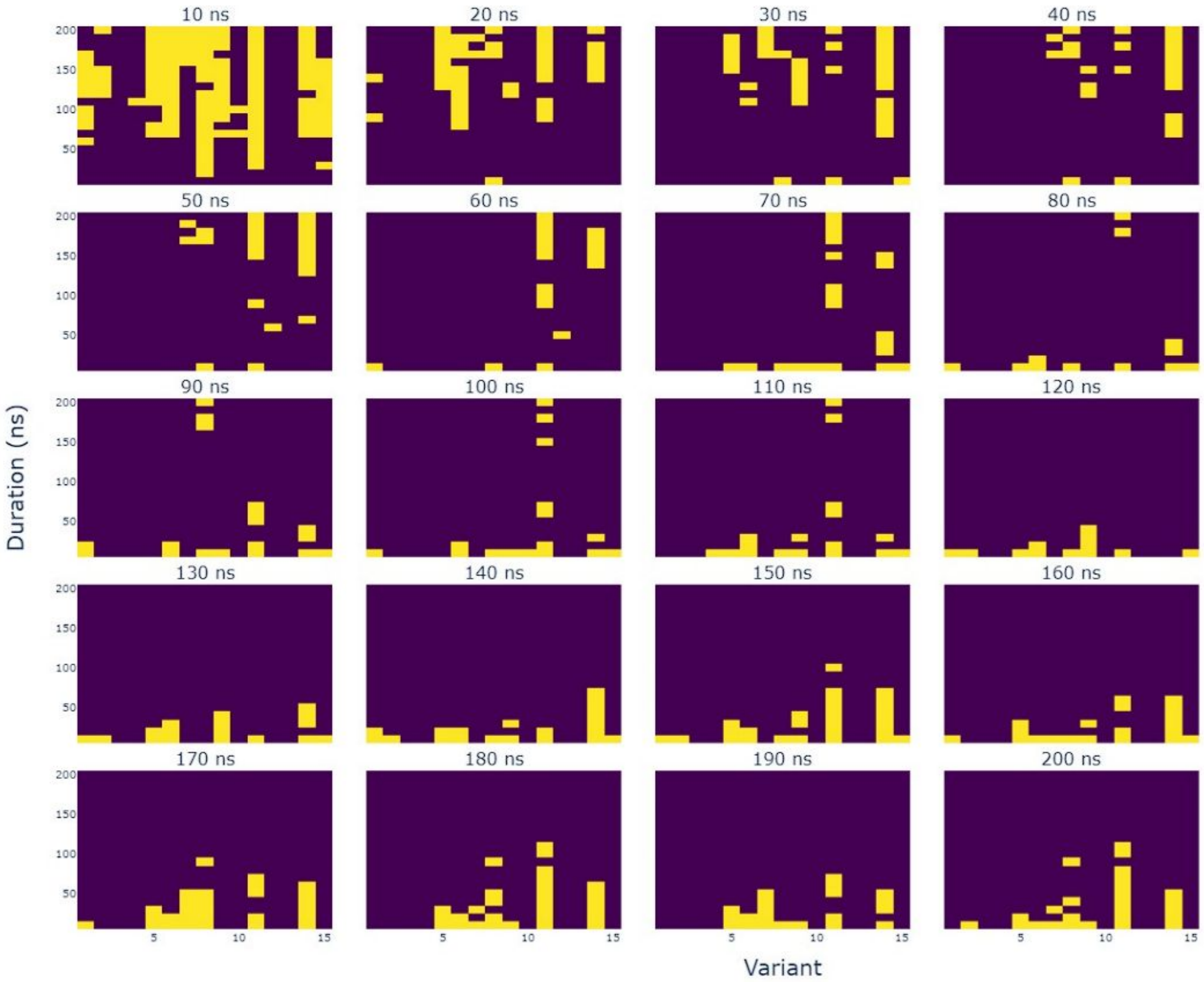
10 ns
20 ns
30 ns
40 ns
50 ns
60 ns
70 ns
80 ns
90 ns
100 ns
110 ns
120 ns
130 ns
140 ns
150 ns
160 ns
170 ns
180 ns
190 ns
200 ns
Duration (ns)
Variant

Figure S2. ANOVA analysis. Heatmaps showing whether any simulation timeframe had statistical difference (yellow) or not (purple) with a threshold of 0.05. On the x-axis we have columns representing the 15 variants and the y-axis shows the different timeframes that the simulation was compared to (e.g., top left the first 10ns timeframe was used and it was compared iteratively to the other timeframes).

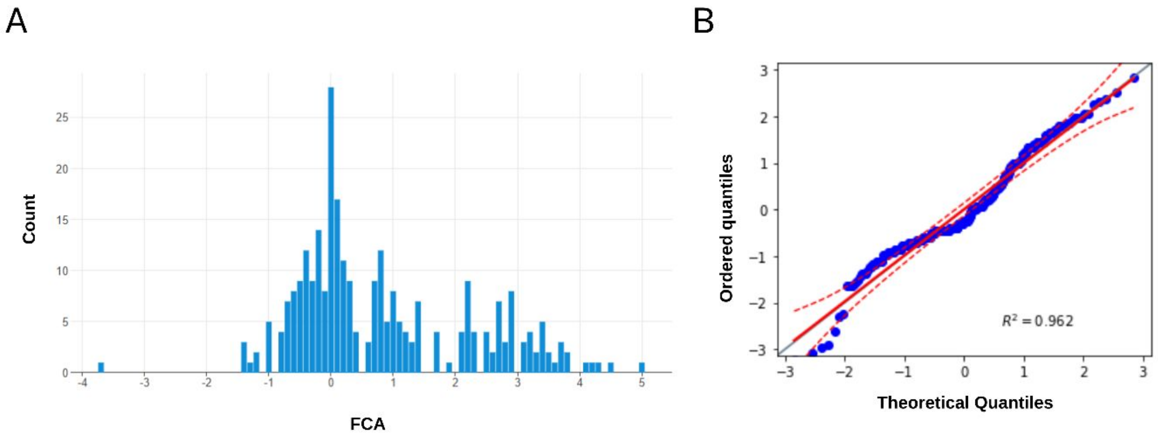

Figure S3. Normality test for the dataset. A) Histogram plot showing the distribution of FCA B) Q-Q plot of FCA.

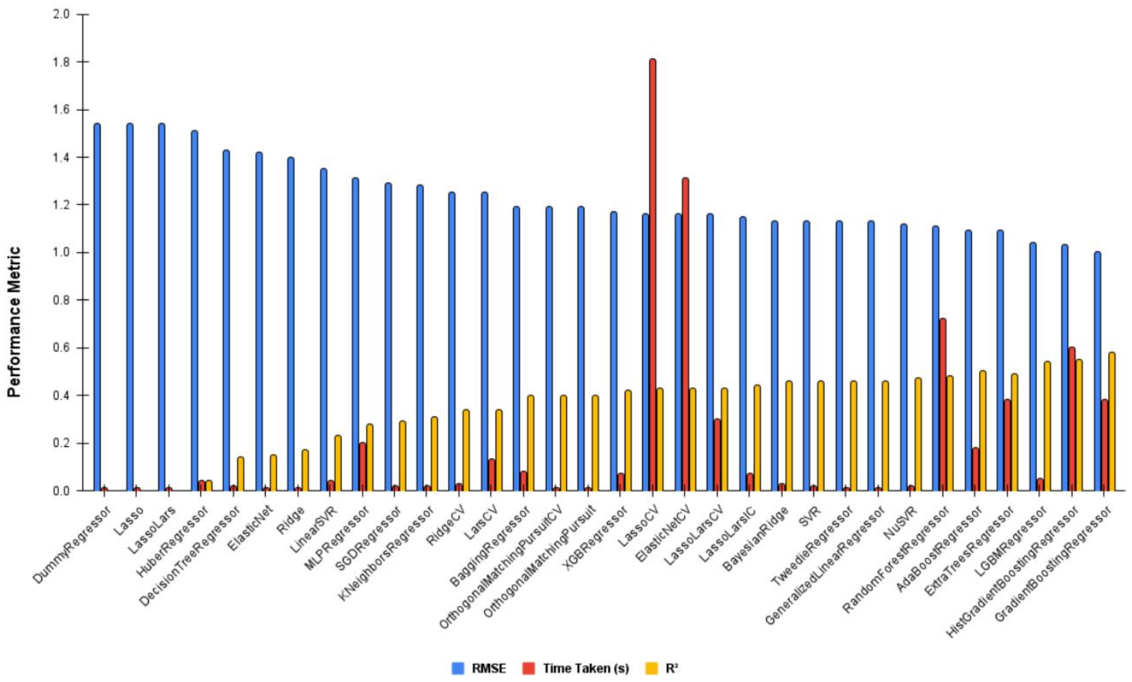

Figure S4. Performance of 32 individual ML models. Individual models or algorithm classes were ranked in the order of decreasing RMSE values shown as blue bars, with low values indicating superior model performance. The time taken for the execution of the model is shown in red, and the goodness of fit (R^2) in yellow.

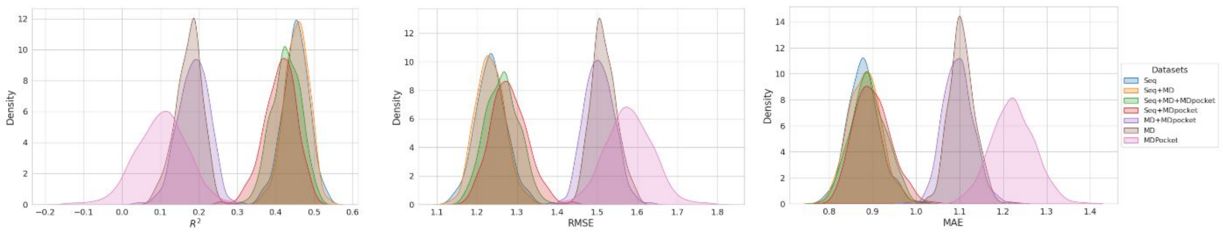

Figure S5. KDE density plot of the 500 boostrapping samples with replacements for each dataset for all three metrics.

Table S1. Raw data of the variants and their corresponding experimental values available at https://zenodo.org/records/10511492.

Table S2. List of features used and their respective acronyms in brackets.

Feature Level	Feature Name	Acronym Used
**Sequence and Structure**	Cruciani's Properties	• Polarity (PP1),<br>• Hydrophobicity (PP2),<br>• H-bonding (PP3).
	Kidera Factors	• Helix/bend preference (KF1),<br>• Side-chain size (KF2),<br>• Extended structure preference (KF3),<br>• Hydrophobicity (KF4),<br>• Double-bend preference (KF5),<br>• Partial specific volume (KF6),<br>• Flat extended preference (KF7),<br>• Occurrence in alpha region (KF8),

		● pK-C (KF9),<br>● Surrounding hydrophobicity (KF10).
	Vectors of Hydrophobic, Steric, and Electronic properties (VHSE)	PCA components of physicochemical variables:<br>● Hydrophobic properties (VHSE1 and VHSE2),<br>● Steric properties (VHSE3 and VHSE4),<br>● Electronic properties (VHSE5 to VHSE8) .
	FASGAI	● Hydrophobicity index (F1),<br>● Alpha and turn propensities (F2),<br>● Bulky properties (F3),<br>● Compositional characteristic index (F4),<br>● Local flexibility (F5),<br>● Electronic properties (F6).
	zScales	● Lipophilicity (Z1),<br>● Steric properties (Steric bulk/Polarizability) (Z2),<br>● Electronic properties (Polarity / Charge) (Z3),<br>● Electronegativity, heat of formation, electrophilicity and hardness (Z4 and Z5).
	tScales	PCA components derived from 67 amino acid descriptors:<br>● First component (T1),

		● Second component (T2),<br>● Third component (T3),<br>● Fourth component (T4),<br>● Fifth component (T5).
	stScales	PCA of 827 structural topological variables:<br>● First component (ST1),<br>● Second component (ST2),<br>● Third component (ST3),<br>● Fourth component (ST4),<br>● Fifth component (ST5),<br>● Sixth component (ST6),<br>● Seventh component (ST7),<br>● Eighth component (ST8).
	protFP	PCA of physicochemical properties:<br>● First component (ProtFP1),<br>● Second component (ProtFP2),<br>● Third component (ProtFP3),<br>● Fourth component (ProtFP4),<br>● Fifth component (ProtFP5),

		• Sixth component (ProtFP6),<br>• Seventh component (ProtFP7),<br>• Eighth component (ProtFP8).
	BLOSUM	BLOSUM indices indicating physiochemical properties:<br>• BLOSUM1,<br>• BLOSUM2,<br>• BLOSUM3,<br>• BLOSUM4,<br>• BLOSUM5,<br>• BLOSUM6,<br>• BLOSUM7,<br>• BLOSUM8,<br>• BLOSUM9,<br>• BLOSUM10.
	MS-WHIM	PCA on 3D of 36 indexes for steric and electrostatic properties:<br>• MSWHIM1,<br>• MSWHIM2,<br>• MSWHIM3.

	BioPython ProtParam	● Isoelectric point (ip),<br>● Molecular weight (molecular.weight),<br>● Aromaticity (aromaticity),<br>● Instability index (instability),<br>● Gravy (gravy),<br>● Percentage for each hydrophobic amino acid (X.g, X.a, X.v, X.l, X.i, X.p, X.f, X.m, X.w).
**MD**	RMSD	All the features here listed are obtained with standard deviation as well (e.g., RMSD_pro_nohand RMSD_pro_noh_st):<br>● RMSD protein no hydrogen (RMSD_pro_noh),<br>● RMSD protein backbone (RMSD_pro_bb),<br>● RMSD protein C-α (RMSD_pro_ca),<br>● RMSD binding site no hydrogen (RMSD_bs_noh),<br>● RMSD binding site backbone (RMSD_bs_bb),<br>● RMSD binding site C-α (RMSD_bs_ca).
	RoG	All the features here listed are obtained with standard deviation as well (e.g., RoG_pro_noh and RoG_pro_noh_sd):<br>● RoG protein no hydrogen (rog_pro_noh),

		● RoG protein backbone (rog_pro_bb),<br>● RoG protein C-α (rog_pro_ca),<br>● RoG binding site no hydrogen (rog_bs_noh),<br>● RoG binding site backbone (rog_bs_bb),<br>● RoG binding site C-α (rog_bs_ca),
	DSSP	● Parallel Beta-sheet (b_para),<br>● Anti-parallel beta-sheet (b_anti),<br>● 3-10 helix (h_3.10),<br>● Alpha helix (halpha),<br>● Pi 3-14 helix (h_pi),<br>● Turn (turn),<br>● Bend (bend),<br>● Random coil (random.coil).
**MDPocket**	MDPocket	All the features here listed are obtained with standard deviation as well (e.g., pocket_volume and pocket_volume_st):<br>● Pocket volume (pocket_volume),<br>● Solvent accessible surface area of the pocket (pocket_asa),

		● Polar solvent accessible surface area of the pocket (pock_pol_asa),<br>● Apolar solvent accessible surface area of the pocket (pock_apol_asa),<br>● Accessible surface area using a probe of 2.2 A instead of 1.4 (pock_asa22),<br>● Polar solvent accessible surface area of the pocket using a probe of 2.2 A instead of 1.4 (pock_pol_asa22),<br>● Apolar solvent accessible surface area of the pocket using a probe of 2.2 A instead of 1.4 (pock_apol_asa22),<br>● Number of alpha spheres (nb_as),<br>● Mean alpha sphere radius (mean_as_ray),<br>● Mean alpha sphere solvent accessibility (mean_as_solv_acc),<br>● Proportion of apolar alpha spheres in the pocket (apol_as_prop),<br>● Mean local hydrophobic density (mean_loc_hyd_dens),<br>● Hydrophobicity score (hydrophobicity_score),<br>● Volume score (volume_score),<br>● Polarity score (polarity_score),<br>● Charge score (charge_score),

		● Maximum distance between the center of mass and all alpha spheres (as_max_dst),<br>● Proportion of polar alpha sphere (Prop_polar_atm),<br>● Nb_abpa,<br>● The volume of the binding pocket measured with convex hull (convex_hull_volume),<br>● Percentage of amino acid type in the binding pocket (three letter code for all 20 canonical amino acids (e.g. ala)).

Table S3. 42 supervised machine learning algorithms along with their brief descriptions.

Model Type	Name of Regressor	Algorithm Description
Dummy	Dummy	A baseline algorithm was utilised to compare to real regressors by always predicting the mean of the training set.
Ensemble	AdaBoost	AdaBoost is a meta-estimator that fits classifiers on the dataset and adjusts its performance by focusing on more difficult cases via weight changes.1 The algorithm version utilised was SAMME.R for faster conversions.2

	Bagging	Bagging regressor is a meta-estimator that works similarly to AdaBoost, however when samples are drawn with replacement.3
	Extra Trees	Extra trees construct trees on randomised subsamples of the dataset and then utilise averages from the decision trees resulting in an improvement in prediction power and minimising over-fitting.4
	Gradient Boosting	Gradient boosting regressor fits in a forward fashion way trees are tuned by taking the derivative of the loss function considering prior predictions.5
	Histogram Gradient Boosting	Histogram Gradient Boosting trees have been developed tailored for large datasets (containing >10000 samples) as this algorithm represents data as bins and allows for faster training and, in instances, better performance.6
	Random Forest	Random Forest regressor builds trees in parallel instead of sequentially allowing for faster computations and implements majority voting instead of pseudo-residual used gradient boosting.7
	Extreme Gradient Boosting	Extreme Gradient Boosting (XGBoost) replicates Gradient Boosting Regressor, however, the algorithm utilises additional regularisation functions that prevent overfitting and manages computational resources better allowing for faster computations.8
	Light Gradient Boosting Machine	Light Gradient Boosting Machine r (LightGBM) shares the same properties as XGBoost, however, in this instance, the trees are built leaf-wise whereas in the latter depth-wise.9
Tree	Decision Tree	Decision Tree is a single tree fit representing the whole dataset.7

	Extra Tree	Extra Tree is the same as Extra Trees Regressor however it is not an ensemble but a single tree.4
Gaussian Process	Gaussian Process	Gaussian Process is a probabilistic regression algorithm, as such provides uncertainty in the measurements.10
Linear Model	Bayesian Ridge	Bayesian Ridge regression uses a flexible regularisation parameter, in this case, Ridge, using priors that adapts to the data.11
	Elastic Net	Elastic Net is a linear model that utilises both Ridge and Lasso regularisation.12
	Elastic Net CV	Elastic Net fit via cross-validation.
	Gamma	Gamma regression is a linear regression model with Gamma distribution.13
	Generalized	Generalized linear regression utilises a link function to link predictions to features.13
	Huber	Huber regression is a linear model using a linear loss onto outliers.14
	Least Angle Regressor (LARS)	LARS is a linear regression with inbuilt forward feature selection that utilises feature correlation with the predictor while fitting.15
	LARS CV	LARS built via cross-validation.
	Lasso	A linear regression with lasso regularisation.16
	Lasso CV	Lasso fit via cross-validation.
	Lasso LARS	A LARS model with Lasso regularisation.17

	Lasso LARS CV	Lasso LARS fit via cross-validation.
	Lasso LARS IC	A Lasso LARS model fit with an information criterion, in this paper, the Akaike information criterion (AIC), a metric for model performance used for model selection.18
	Linear Regression	A simple linear regression.19
	Orthogonal Matching Pursuit (OMP)	OMP is an algorithm that minimises the noise in the dataset via applying constraints on non-zero coefficients.20
	OMP CV	OMP fit via cross-validation.
	Passive Aggressive	Passive Aggressive Regression is an online ML algorithm that builds regression incrementally via an increase of instances used for training.21
	Poisson	Poisson regression is a linear regression model with Poisson distribution.22
	RANdom SAmple Consensus (RANSAC)	RANSAC is a linear model that utilises subsets of inliers for the model construction.23
	Ridge	A linear regression with Ridge regularisation.24
	Ridge CV	Ridge fit via cross-validation.

	Stochastic Gradient Descent (SGD)	A linear model fitted by minimizing the cost function through stochastic gradient descent.25
	Transformed Target	A linear regression that allows transformations on the target variable.26
	Tweedie	Tweedie regression is a linear regression model with Tweedie distribution.
Clustering	K-Neighbors	A linear model that utilises local neighbours for predictions.27
Neural Network	Multi-Layer Perceptron	A supervised neural network regression.28
Kernel	Support Vector Regression (SVR)	A support vector regression tuned via epsilon and C regularisation.29
	Nu SVR	A regressor that tunes the numbers of vectors by the parameter Nu.29
	Linear SVR	Similar to SVR however built with linear regularisations.29
	Kernel Ridge	Kernel Ridge is a ridge regression kernelized. A linear kernel was utilised in this paper.29,30

Table S4. Random Search Space for the selection of the best LightGBM hyperparameters for the main dataset and the data subsets.

Parameter	Searched Space	Selected Parameter
Learning Rate	From 0.001 to 1, step size of 0.002	0.010
Maximum Depth	From 1 to 100, step size of 10	51
Number of leaves	From 5 to 100, step size of 5	61
Minimum split gain	From 0 to 20, step size of 1	0
Colum sample by tree	From 0.1 to 1, step size of 0.2	1
Numbers of estimators	From 10 to 1000, step size of 10	280
Alpha regularisation factor	From 0.1 to 1, step size of 0.1	0.1
Lamba regularisation factor	From 1 to 10, step size of 1	6

Table S5. Complete dataset of the features extracted and used for the modelling and analysis presented in the paper available at https://zenodo.org/records/10511492.

Table S6. Best RandomizedSearchCV Hyperparameters Identified for each dataset.

Dataset	Best RandomizedSearchCV Hyperparameters
Seq	colsample_bytree=0.8, learning_rate=0.008, max_depth=41,

	min_split_gain=1, n_estimators=520, num_leaves=42, random_state=3031, reg_alpha=0.6, reg_lambda=7
Seq+MD	colsample_bytree=0.8, learning_rate=0.008, max_depth=41, min_split_gain=1, n_estimators=520, num_leaves=42, random_state=3031, reg_alpha=0.6, reg_lambda=7
Seq+MD+MDpocket	colsample_bytree=0.8, learning_rate=0.008, max_depth=41, min_split_gain=1, n_estimators=520, num_leaves=42, random_state=3031, reg_alpha=0.6, reg_lambda=7
Seq+MDpocket	colsample_bytree=0.8, learning_rate=0.008, max_depth=41, min_split_gain=1, n_estimators=520, num_leaves=42, random_state=3031, reg_alpha=0.6, reg_lambda=7
MD+MDpocket	colsample_bytree=0.8, learning_rate=0.04, max_depth=91, min_split_gain=8, n_estimators=830, num_leaves=42, random_state=3031, reg_alpha=0.8, reg_lambda=5
MD	colsample_bytree=0.8, learning_rate=0.04, max_depth=91, min_split_gain=8, n_estimators=830, num_leaves=42, random_state=3031, reg_alpha=0.8, reg_lambda=5

MDpocket	colsample_bytree=0.8, learning_rate=0.008, max_depth=41, min_split_gain=1, n_estimators=520, num_leaves=42, random_state=3031, reg_alpha=0.6, reg_lambda=7

Table S7. Permutation Feature Importance best 10 ranking features across the datasets.

**Rank**	**Seq**	**Seq+ MD**	**Seq+MD+MDpocket**	**Seq+MD pocket**	**MD+MD pocket**	**MD**	**MDpocket**
1	BLOSUM8	VHSE2	VHSE2	BLOSUM8	halpha	halpha	arg
2	VHSE2	BLOSUM8	halpha	VHSE2	arg	h_3.10	his_st
3	KF7	halpha	BLOSUM8	KF7	arg_st	bend	arg_st
4	ST2	Z3	T4	arg_st	turn	rmsd_bs_bb	apol_as_prop
5	Z3	KF8	ST2	ST2	bend	turn	charge_score
6	BLOSUM2	h_3.10	KF8	arg	apol_as_prop	rmsd_pro_ca	mean_as_solv_acc_st
7	MSWHIM1	KF7	KF7	BLOSUM2	his_st	rog_bs_noh	gly
8	KF8	T4	MSWHIM1	ProtFP8	rmsd_pro_ca	rmsd_bs_ca	as_max_dst_st

9	KF10	BLOSUM2	rmsd_pro_noh_sd	his_st	h_3.10	rmsd_bs_noh	phe_st
10	ProtFP8	ST2	ProtFP1	T4	rmsd_bs_ca	rog_pro_bb	convex_hull_volume_st

References

(1) Freund, Y.; Schapire, R. E. A Decision-Theoretic Generalization of On-Line Learning and an Application to Boosting. J Comput Syst Sci 1997, 55 (1), 119–139. https://doi.org/10.1006/jcss.1997.1504.

(2) Hastie, T.; Rosset, S.; Zhu, J.; Zou, H. Multi-Class AdaBoost. Stat Interface 2009, 2 (3), 349–360. https://doi.org/10.4310/sii.2009.v2.n3.a8.

(3) Ho, T. K. The Random Subspace Method for Constructing Decision Forests. Ieee T Pattern Anal 2021, 20 (8), 832–844. https://doi.org/10.1109/34.709601.

(4) Geurts, P.; Ernst, D.; Wehenkel, L. Extremely Randomized Trees. Mach Learn 2006, 63 (1), 3–42. https://doi.org/10.1007/s10994-006-6226-1.

(5) Friedman, J. H. Greedy Function Approximation: A Gradient Boosting Machine. Ann Statistics 2001, 29 (5). https://doi.org/10.1214/aos/1013203451.

(6) Guryanov, A. Analysis of Images, Social Networks and Texts, 8th International Conference, AIST 2019, Kazan, Russia, July 17–19, 2019, Revised Selected Papers. Lect Notes Comput Sc 2019, 39–50. https://doi.org/10.1007/978-3-030-37334-4_4.

(7) Breiman, L.; Friedman, J. H.; Olshen, R. A.; Stone, C. J. Classification And Regression Trees. 2017. https://doi.org/10.1201/9781315139470.

(8) Chen, T.; Guestrin, C. XGBoost: A Scalable Tree Boosting System. Arxiv 2016. https://doi.org/10.1145/2939672.2939785.

(9) Ke, G.; Meng, Q.; Finley, T.; Wang, T.; Chen, W.; Ma, W.; Ye, Q.; Liu, T.-Y. LightGBM: A Highly Efficient Gradient Boosting Decision Tree. Advances in neural information processing systems 2017, 30, 3146–3154.

(10) Seeger, M. Gaussian Processes for Machine Learning. Int J Neural Syst 2003, 14 (2), 69–106. https://doi.org/10.1142/s0129065704001899.

(11) MacKay, D. J. C. Bayesian Interpolation. Neural Comput 1992, 4 (3), 415–447. https://doi.org/10.1162/neco.1992.4.3.415.

(12) Zou, H.; Hastie, T. Regularization and Variable Selection via the Elastic Net. J Royal Statistical Soc Ser B Statistical Methodol 2005, 67 (2), 301–320. https://doi.org/10.1111/j.1467-9868.2005.00503.x.

(13) Nelder, J. A.; Wedderburn, R. W. M. Generalized Linear Models. J Royal Statistical Soc Ser Gen 1972, 135 (3), 370–384. https://doi.org/10.2307/2344614.

(14) Huber, P. J.; Ronchetti, E. M. Robust Statistics; Wiley, Ed.; 2nd, Series Ed.; Wiley, 2009.

(15) Efron, B.; Hastie, T.; Johnstone, I.; Tibshirani, R. Least Angle Regression. Ann Statistics 2004, 32 (2). https://doi.org/10.1214/009053604000000067.

(16) Tibshirani, R. Regression Shrinkage and Selection Via the Lasso. J Royal Statistical Soc Ser B Methodol 1996, 58 (1), 267–288. https://doi.org/10.1111/j.2517-6161.1996.tb02080.x.

(17) Fraley, C.; Hesterberg, T. Least Angle Regression and LASSO for Large Datasets. Statistical Analysis Data Min Asa Data Sci J 2009, 1 (4), 251–259. https://doi.org/10.1002/sam.10021.

(18) Zou, H.; Hastie, T.; Tibshirani, R. On the "Degrees of Freedom" of the Lasso. Ann Statistics 2007, 35 (5), 2173–2192. https://doi.org/10.1214/009053607000000127.

(19) Galton, F. Regression Towards Mediocrity in Hereditary Stature. J Anthropological Inst Gt Br Irel 1886, 15, 246. https://doi.org/10.2307/2841583.

(20) Mallat, S. G.; Zhang, Z. Matching Pursuits with Time-Frequency Dictionaries. Ieee T Signal Proces 1993, 41 (12), 3397–3415. https://doi.org/10.1109/78.258082.

(21) Crammer, K.; Dekel, O.; Keshet, J.; Shalev-Shwarts, S.; Singer, Y. Online Passive-Aggressive Algorithms. JMLR 2006, 551–585.

(22) Tutz, G. International Encyclopedia of Statistical Science. 2014, 1075–1077. https://doi.org/10.1007/978-3-642-04898-2_450.

(23) Fischler, M. A.; Bolles, R. C. Random Sample Consensus: A Paradigm for Model Fitting with Applications to Image Analysis and Automated Cartography. Commun Acm 1981, 24 (6), 381–395. https://doi.org/10.1145/358669.358692.

(24) Obenchain, R. L. Book Review: Improving Efficiency by Shrinkage: The James-Stein and Ridge Regression Estimators. Stat Methods Med Res 1999, 8 (4), 333–334. https://doi.org/10.1177/096228029900800408.

(25) Kiefer, J.; Wolfowitz, J. Stochastic Estimation of the Maximum of a Regression Function. Ann Math Statistics 1952, 23 (3), 462–466. https://doi.org/10.1214/aoms/1177729392.

(26) Bonat, W. H.; Kokonendji, C. C. Flexible Tweedie Regression Models for Continuous Data. J Stat Comput Sim 2017, 87 (11), 1–15. https://doi.org/10.1080/00949655.2017.1318876.

(27) Yao, Z.; Ruzzo, W. L. A Regression-Based K Nearest Neighbor Algorithm for Gene Function Prediction from Heterogeneous Data. Bmc Bioinformatics 2006, 7 (Suppl 1), S11. https://doi.org/10.1186/1471-2105-7-s1-s11.

(28) Wilusz, T. Neural Networks — A Comprehensive Foundation. Neurocomputing 1995, 8 (3), 359–360. https://doi.org/10.1016/0925-2312(95)90026-8.

(29) Platt, J. C. Probabilistic Outputs for Support Vector Machines and Comparisons to Regularized Likelihood Methods. Advances in large margin classifiers 1999, 10.3, 61–74.

(30) Murphy, K. P. Machine Learning: A Probabilistic Perspective; Press, M., Ed.; Press, M., Series Ed.; 1970.
